# Toll-like receptors in cerebral ischemic inflammatory injury

**DOI:** 10.1186/1742-2094-8-134

**Published:** 2011-10-08

**Authors:** Yan-Chun Wang, Sen Lin, Qing-Wu Yang

**Affiliations:** 1 Department of Neurology, Daping Hospital, Third Military Medical University, Changjiang Branch Road No. 10, Yuzhong District, Chongqing 400042, PR China; 2 Development and Regeneration Key Laboratory of Sichuan Province, Department of Histo-embryology and Neurobiology, Chengdu Medical College, Chengdu 610083, PR China

**Keywords:** cerebral ischemia, Toll-like receptors (TLRs), inflammation, innate immunity

## Abstract

Cerebral ischemia triggers acute inflammation, which has been associated with an increase in brain damage. The mechanisms that regulate the inflammatory response after cerebral ischemia are multifaceted. An important component of this response is the activation of the innate immune system. However, details of the role of the innate immune system within the complex array of mechanisms in cerebral ischemia remain unclear. There have been recent great strides in our understanding of the innate immune system, particularly in regard to the signaling mechanisms of Toll-like receptors (TLRs), whose primary role is the initial activation of immune cell responses. So far, few studies have examined the role of TLRs in cerebral ischemia. However, work with experimental models of ischemia suggests that TLRs are involved in the enhancement of cell damage following ischemia, and their absence is associated with lower infarct volumes. It may be possible that therapeutic targets could be designed to modulate activities of the innate immune system that would attenuate cerebral brain damage. Ischemic tolerance is a protective mechanism induced by a variety of preconditioning stimuli. Interpreting the molecular mechanism of ischemic tolerance will open investigative avenues into the treatment of cerebral ischemia. In this review, we discuss the critical role of TLRs in mediating cerebral ischemic injury. We also summarize evidence demonstrating that cerebral preconditioning downregulates pro-inflammatory TLR signaling, thus reducing the inflammation that exacerbates ischemic brain injury.

## Introduction

Cerebral ischemia, the most common cerebrovascular disease, is one of the leading causes of morbidity and mortality around the world. However, many details of the pathogenesis of cerebral ischemia are not fully known. Cerebral ischemia is a condition of complex pathology that includes several inflammatory events, such as aggregation of inflammatory cells and upregulation of cytokines. Particularly, accumulating evidence suggests that Toll-like receptors (TLRs) are important mediators of cerebral ischemic injury. Therefore, understanding TLRs and their relationship to cerebrovascular disease is becoming increasingly important to basic and clinical scientists.

TLRs are key receptors in the mammalian innate immune response to infectious microorganisms, but are also activated by host-derived molecules. The association between TLRs and the activation of a variety of downstream inflammatory cascades has been established in cerebral ischemia, as well as an involvement in inflammatory injury. Additionally, many diverse neuroprotective networks may redirect TLR signaling as one mechanism of endogenous protection.

The purpose of this review is to (1) summarize current knowledge on TLR signaling; (2) examine the evidence implicating TLRs in cerebral ischemia injury, (3) outline known mechanisms of TLR-mediated neuronal damage, and (4) summarize the information on other molecules involved in TLR signaling. The latter may help identify potential clinical targets for preventing TLR-mediated cerebral ischemic injury.

### The innate immune response in the central nervous system (CNS)

It was initially believed that innate immunity was an immunological program engaged by peripheral organs to maintain homeostasis after nonspecific stress and injury. It has now been long documented that innate immunity is a highly organized response that also takes place in the CNS [1,2]. In fact, the CNS shows a well-organized innate immune reaction in response to systemic bacterial infection and cerebral injury [1,3].

The innate immune response in the CNS is characterized by the expression of various immunological proteins in the circumventricular organs as well as other structures that are not subject to the blood-brain barrier (BBB). This expression of immunological proteins extends progressively to affect microglia across the brain parenchyma and may lead to the onset of an adaptive immune response. The innate immune system of the CNS maintains a critical balance between the protective and the potentially harmful effects of its activation following acute brain injury, the so-called "double-edged sword" effect [4]. The balance between the destructive and protective effects of the innate immune response must be precisely regulated to promote conditions that support brain repair and maintain tissue homeostasis [5].

The innate immune response of the CNS relies upon its resident cells' (neurons and glia) phagocytic and scavenger receptors, which are capable of distinguishing "self" from "nonself " [6]. Microglia, the resident immune cells of the CNS, are sensitive sensors of events occurring within their environment and provide the first line of defense against invading microbes [6]. Microglia respond to CNS injuries with increased proliferation, motility, phagocytic activity, and the release of cytokines and reactive oxygen species [7]. Upon recognition of pathogens, activated microglia accumulate at sites of tissue damage and express proinflammatory cytokines, adhesion molecules, and free radicals [2,8]. Activation of microglia also results in increased expression of major histocompatibility complex and co-stimulatory molecules, and stimulates responses in CD4 and CD8 T helper cells. Therefore microglia serve as important antigen-presenting cells of the CNS [7].

CNS injuries also trigger phagocytic and cytotoxic functions in microglia. When activated, microglia upregulate opsonic receptors. These include both complement (CR1, CR3, CR4) and Fcγ receptors (I, II, III), which enhance phagocytic activity by binding to complement components and immunoglobulin fragments, respectively [7]. In contrast, the cytotoxic functions of microglia are carried out through the release of superoxide radicals and proinflammatory mediators into the microenvironment in response to pathogens and cytokine stimulation [7]. It has also been noted that microglia are activated in some diseases of the CNS, they are among the first cells found at the site of tissue injury and infection, and recruit other immune cells [2]. Therefore, microglia play a central role in innate immunity, recognizing both pathogen- and damage-associated molecular patterns, and have been implicated in a range of neuronal inflammatory processes.

### Toll-like receptors (TLRs) in CNS

In the past few years, it has become evident that the innate immune system, and in particular pattern recognition receptors, have evolved to detect components of foreign pathogens. These components are referred to as pathogen-associated molecular patterns (PAMPs), and include Toll-like receptors (TLRs) which play a major role in both infectious and non-infectious CNS diseases [9-11].

TLRs are type I transmembrane proteins with ectodomains containing leucine-rich repeats. These repeats mediate the recognition of PAMPs, transmembrane domains, and intracellular Toll-interleukin 1 (IL-1) receptor (TIR) domains required for downstream signal transduction [11]. So far, 10 and 12 functional TLRs have been identified in humans and mice, respectively, with TLR1-TLR9 being conserved in both species. Mouse TLR10 is not functional because of a retrovirus insertion, and TLR11, TLR12 and TLR13 have been lost from the human genome [10].

Studies of mice deficient in each TLR have demonstrated that each TLR has a distinct function in terms of PAMP recognition and immune responses [10]. PAMPs recognized by TLRs include lipids, lipoproteins, proteins and nucleic acids derived from a wide range of microbes such as bacteria, viruses, parasites and fungi [10]. The recognition of PAMPs by TLRs occurs in various cellular compartments, including the plasma membrane, endosomes, lysosomes and endolysosomes [10]. TLRs detect a wide range of PAMPs that are found on bacteria, viruses, fungi, and parasites. These include proteins, lipids, and nucleic acids. For example, TLRs recognize the bacterial cell wall components peptidoglycan (TLR2) and lipopolysaccharide (TLR4), as well as dsRNA (TLR3), ssRNA (TLR7), and non-methylated cytosine-guanosine (CpG) DNA (TLR9) [9,10].

### TLR expression in the CNS

Constitutive expression of TLRs within the brain occurs in microglia and astrocytes, and is largely restricted to the circumventricular organs and meninges areas with direct access to the circulation [12]. In general, TLRs are located on antigen-presenting cells such as B cells, dendritic cells, monocytes, macrophages, and microglia in the CNS. In addition, these receptors can be expressed by the endothelium and by cells within the brain parenchyma such as astrocytes, oligodendrocytes, and neurons [13,14]. For example, human microglia express TLRs 1-9 and generate cytokine profiles tailored by the specific TLR stimulated [13,15]. Similarly, human astrocytes express TLRs 1-9, with particularly prominent TLR3 expression [15].

Oligodendrocytes and endothelial cells express a relatively limited repertoire of TLRs. Oligodendrocytes express TLRs 2 and 3, while cerebral endothelial cells constitutively express TLRs 2, 4, and 9 and increase their expression of these TLRs in response to stressful stimuli [15]. Human neurons express TLRs 2, 3, 4, 8, and 9 [15].

Notably, microglia and astrocytes respond differently to specific TLR engagement, reflective of their distinct roles in the brain. Microglia initiate robust cytokine and chemokine responses upon stimulation of TLR2 (TNF-α, IL-6, IL-10), TLR3 (TNF-α, IL-6, IL-10, IL-12, CXCL-10, IFN-β), and TLR4 (TNF-α, IL-6, IL-10, CXCL-10, IFN-β), yet astrocytes initiate only minor IL-6 responses to all but TLR3 stimulation [12].

### TLR signaling

**Figure 1:**
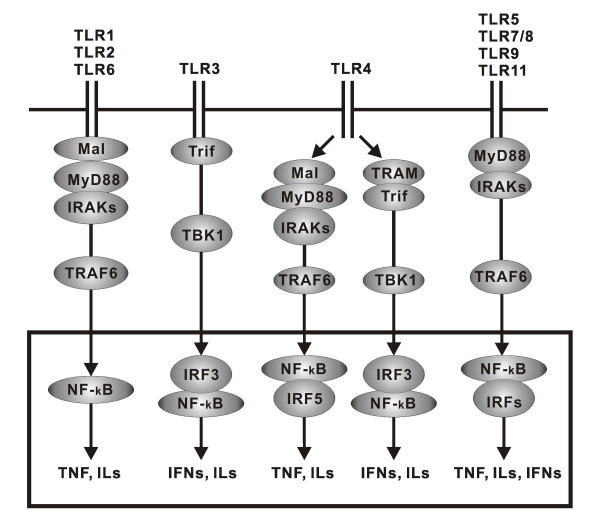
**Toll-like receptor (TLR) signaling**. TLRs are transmembrane proteins with a large extra-cellular domain containing a cytoplasmic Toll/IL-1 receptor (TIR) domain. All TLR family members, except TLR3, signal through the myeloid differentiation primary-response gene 88 (MyD88) to recruit downstream interleukin (IL)-1 receptor-associated kinases (IRAKs) and tumor necrosis factor (TNF)-receptor associated factor 6 (TRAF6). In TLR2 and TLR4 signaling, MyD88 adaptor-like protein (MAL) is required for recruiting MyD88 to their receptors, whereas in others such as TLR5, TLR7, TLR9, and TLR11, MAL is not required. TLR1 and TLR2 or TLR2 and TLR6 form heterodimers that signal through MAL/MyD88. TLR3 signals through the adaptor TIR-domain-containing adaptor protein inducing interferon (IFN)-β-mediated transcription-factor (Trif), which recruits and activates TNF receptor-associated factor-family member-associated NF-κB activator-binding kinase 1 (TBK1). In addition to the MAL/MyD88-dependent pathway, TLR4 can also signal through a MyD88-independent pathway that activates TBK1 via a Trif-related adaptor molecule (TRAM)-Trif-dependent mechanism. TLR5, TLR7/8, TLR9, and TLR11 use only MyD88 as its signaling adaptor. These kinases ultimately activate transcription factors such as nuclear factor-κB (NF-κB) and IFN regulatory factors (IRFs), which result in production of various cytokines such as TNF, IL, and IFNs.

The TLRs signal through common intracellular pathways leading to transcription factor activation and the generation of cytokines and chemokines (Figure 1) [16]. TLRs recruit five adaptors including myeloid differentiation primary response gene 88 (MyD88), MyD88 adaptor-like protein (MAL), TIR-domain-containing adaptor protein inducing interferon (IFN)-β-mediated transcription factor (TRIF), TRIF-related adaptor molecule (TRAM), and sterile α- and armadillo motif-containing protein (SARM) [17]. TLRs interact with their respective adaptors via the homologous binding of their unique TIR domains present in both the receptors and the adaptor molecules.

Based on the specific adaptors recruited, TLR signaling can take either the MyD88-dependent or MyD88-independent pathways. In general, each TLR family member, with the exception of TLR3, signals through the MyD88-dependent pathway, initiated by the MyD88 adaptor protein. Recruitment of MyD88 to the activated receptor initiates formation of the IL-1 receptor associated kinase (IRAK) complex resulting in phosphorylation of IKKα/β, activation of the transcription factors NF-κB, interferon-β promoter-binding protein (IRF)1, and IRF7, and generation of the pro-inflammatory cytokines IL-6 and TNF-α, among others [18].

TLR3, on the other hand, signals through the MyD88-independent pathway, initiated by the TRIF adaptor molecule. Recruitment of TRIF to the receptor initiates phosphorylation of IKKε, which activates the transcription factors IRF3 and IRF7, and generates anti-viral molecules such as IFN-β. Of the TLRs, only TLR4 can utilize either of these pathways [18].

It is noteworthy that MyD88 is also recruited to the endosomal receptors TLR7 and TLR9, again enlisting members of the IRAK family [11]. Due to the endosomal location of the complex, the phosphorylated IRAKs are able to bind TRAF3 in addition to TRAF6. Activation of TRAF3 leads to phosphorylation, dimerization, and nuclear localization of the transcription factors IRF3, IRF5, and IRF7 with resultant type I IFN production. Hence these endosomal TLRs are capable of signaling to NF-κB, AP-1 and IRFs, resulting in a diverse genomic response [11].

### TLR ligands

TLRs are largely divided into two subgroups depending on their cellular localization and respective PAMP ligands. One group is composed of the TLRs 1, 2, 4, 5, 6 and 11, which are expressed on cell surfaces and recognize mainly microbial membrane components such as lipids, lipoproteins, and proteins. The other group consists of TLRs 3, 7, 8 and 9, which are expressed exclusively in intracellular vesicles such as the endoplasmic reticulum (ER), endosomes, lysosomes and endolysosomes, where they recognize microbial nucleic acids [15] (Table 1).

**Table 1:** Exogenous and endogenous TLR ligands.

TLRs	Major cell types	Exogenous ligands	Endogenous ligands
TLR1	Myeloid cells T, B and NK cells, microglia, astrocytes	Bacterial triacyl-lipopeptide	
TLR2	Myeloid cells, T cells, microglia, astrocytes, oligodendrocytes, neurons	Lipoproteins/lipopeptides, lipoteichoic acid, lipoarabinomannan, peptidoglycan, glycoinositolphospholipids, glycolipids, porins, zymosan, atypical lipopolysaccharide	Heat-shock proteins 60 and 70, Gp96, Saturated fatty acids
TLR3	Epithelial cells, dendritic cells, microglia, astrocytes, oligodendrocytes, neurons	Double-stranded RNA	mRNA
TLR4	Myeloid cells, microglia, astrocytes, neurons	Lipopolysaccharide, paclitaxel, respiratory syncytial virus fusion protein, mouse mammary tumor virus envelope proteins	Heat-shock proteins 60 and 70, Gp96, Type III repeat extra domain A of fibronectin, oligosaccharides of hyaluronic acid, polysaccharide fragments of heparin sulfate, fibrinogen, high mobility group box 1, surfactant protein-A, β-defensin 2
TLR5	Myeloid cells, epithelial cells, microglia, astrocytes	Flagellin	
TLR6	Myeloid cells, dendritic cells, microglia, astrocytes	Phenol-soluble modulin, diacyl lipopeptides, lipoteichoic acid, zymosan	
TLR7	B cells, dendritic cells, microglia, astrocytes	Imidazoquinoline, loxoribine, bropirimine, Single-stranded RNA	
TLR8	Myeloid cells, microglia, astrocytes, neurons	Single-stranded RNA	
TLR9	Epithelial and B cells, dendritic cells, microglia, astrocyte, neuron	Unmethylated CpG DNA	Chromatin-IgG complexes
TLR10	B cells, dendritic cells	Unknown, may interact with TLR2	
TLR11	Myeloid cells, uroepithelial cells	Uropathogenic *E. coli*	

(Marsh et al., 2009b[13];Takeda and Akira, 2004[18]; Cristofaro and Opal, 2006[67]; Guo and Schluesener, 2007[68]; Tsan and Gao, 2004[69];)

In detail, TLR4 predominantly recognizes lipopolysaccharide (LPS) from gram-negative bacteria. TLR2 dimerizes with TLR1 to recognize triacylated lipopeptides from bacteria. TLR2 also dimerizes with TLR6 and responds to a variety of PAMPs including peptidoglycans, diacylated lipopeptides such as Pam2CSK4, LPSs of gram-positive bacteria, fungal zymosan, and mycoplasma lipopeptides. TLR5 is mainly expressed in the intestine where it senses bacterial flagellin protein. TLR11 possibly recognizes an unknown ligand from an uropathogenic bacteria and a profiling-like molecule of the protozoan *Toxoplasma gondii*. TLR3 is activated in response to double-stranded RNA (dsRNA) of viral origin. Human TLR8 and its murine orthologue, TLR7, recognize imidazoquinoline and viral ssRNA. TLR9 recognizes unmethylated CpG dinucleotides found in bacteria as well as viral genomes.

TLRs also detect some endogenous ligands, including fibrinogen, heat shock proteins (HSP; HSP60, and HSP70 for TLR2 and 4), saturated fatty acids (TLR 2 and 4), mRNA (TLR3), hyaluronan fragments, heparan sulfate, fibronectin extra domain A, lung surfactant protein A, or high mobility group box 1 protein (HMGB1; TLR4). The known endogenous ligands of TLRs are either molecules released from damaged cells or extracellular matrix breakdown products. In this way, innate immune inflammatory responses may be activated without the presence of invading pathogens but merely as a result of tissue damage.

### TLRs and ligands in cerebral ischemic damage

Accumulating evidence shows that ischemic injury and inflammation account for the pathogenic progression of stroke [15,19,20]. The distal cascade of inflammatory responses that result in organ damage after ischemic injury has been studied extensively. The ability of TLRs to mediate inflammatory responses in immune cells suggests their involvement in these and in ischemia-induced brain damage.

The inflammatory response to cerebral ischemia is initiated by the detection of injury-associated molecules by local cells such as microglia and astrocytes. The response is further promoted by infiltrating neutrophils and macrophages, resulting in the production of inflammatory cytokines, proteolytic enzymes, and other cytotoxic mediators [13]. Recent reports provide evidence that TLRs and their ligands play a crucial role in cerebral ischemic injuries and neuronal cell death [19-30]. However, the complex array of mechanisms and the precise role of TLRs in mediating neuronal damage remain to be fully elucidated.

### The role of TLR4 in cerebral ischemia

TLR4 plays an important role in the innate immunity of the CNS [31]. Numerous studies demonstrate that TLR4 participates in cerebral injury upon ischemic stroke. Several studies confirm that cerebral ischemia results in the upregulation of *TLR4 *mRNA in neurons as early as one hour after initiation of ischemia *in vivo *[19,32].

Importantly, cortical neuronal cultures from TLR4-deficient mice show increased survival after glucose deprivation [32]. Mice lacking TLR4 exhibit reduced infarct size compared with wild-type mice after cerebral ischemic injury [23,24,32-34]. TLR4-mutant mice subjected to middle cerebral artery occlusion (MCAO) or animals suffering global cerebral ischemia exhibit improved neurological behavior and reduced edema, as well as reduced levels of secretion of proinflammatory cytokines such as TNF-α and IL-6 [23,24,33]. In addition, mice lacking TLR4 have reduced expression of inducible nitric oxide synthase (iNOS), cyclooxygenase 2 (COX2), and IFN-γ [24,33].

Likewise, a TLR4 mutation confers protection against MCAO [34]. Moreover, after MCAO, loss of TLR4 function is associated with reduced expression of p38 and Erk1/2 in damaged neurons, implicating TLR4 in MCAO injury [23,24,32,34].

Taken together, these studies indicate that TLR4 signaling modulates the severity of ischemia-induced neuronal damage.

### The role of TLR2 in cerebral ischemia

TLR2 has been shown to play a role in cerebral ischemic damage [32,35-38]. *TLR2 *mRNA was upregulated in the brain of mice during cerebral ischemia and expressed in lesion-associated microglia [32]. TLR2-deficient mice displayed less CNS injury compared with wild-type mice in a model of focal cerebral ischemia [32]. Neurons from TLR2-knockout mice were protected against cell death induced by energy deprivation [35]. And, the amount of brain damage and neurological deficits caused by a MCAO were significantly less in mice deficient in TLR2 compared with wild-type control mice [35]. Moreover, TLR2 has been proved to be the most significantly upregulated TLR in the ipsilateral brain hemisphere [36].

TLR2 protein was expressed mainly in microglia in post-ischemic brain tissue, but also in selected endothelial cells, neurons, and astrocytes; TLR2-related genes with pro-inflammatory and pro-apoptotic capabilities were also induced. Two days after a one hour induction of transient focal cerebral ischemia, the infarct volume in TLR2-deficient mice was significantly smaller compared to wild-type mice. Therefore, TLR2 upregulation and TLR2 signaling are important events in focal cerebral ischemia and contribute to ischemic damage [36].

Interestingly, one recent study demonstrated that inflammatory signaling of the TLR2 heterodimer TLR2/1 in the post-ischemic brain requires the scavenger receptor CD36 [37]. In CD36-null mice, activators of TLR2/1 did not trigger inflammatory gene expression and did not exacerbate ischemic injury. The link between CD36 and TLR2/1 was specific for brain inflammation because CD36 is required for TLR2/6 (another TLR2 heterodimer) signaling. These findings raise the possibility that the TLR2/1-CD36 complex is a critical sensor of danger signals produced by cerebral ischemia [37].

A more recent study demonstrated that TLR2 mediates leukocyte and microglial infiltration and neuronal death, which can be attenuated by TLR2 inhibition [38]. The TLR2 inhibition *in vivo *improves neuronal survival and may represent a future stroke therapy [38].

However, studies have demonstrated that TLR2 and TLR4 appear to play opposing roles in cerebral ischemia [35,36,39]. Ziegler et al compared the response of TLR2^-/- ^and TLR4^-/- ^mice to cerebral ischemia [36]. They found that TLR2^-/- ^mice had a smaller infarct size. However, Hua et al. demonstrated that brain infarct size was significantly less in TLR4^-/- ^mice but was increased in TLR2^-/- ^mice [39]. The difference between this study and that of Ziegler et al. may be because Zeigler et al. occluded the middle cerebral artery, whereas Hua et al. occluded the common and internal carotid arteries. Alternatively, the difference in results may be a consequence of the differing genetic backgrounds of the transgenic mice.

### The role of HMGB1 in cerebral ischemia

The TLR endogenous ligand HMGB1 has been very recently implicated in the mechanism of ischemic brain damage [21,25-28,40,41]. Three novel studies in particular have indicated that HMGB1 plays a pivotal role in ischemic brain injury. Firstly, short hairpin RNA (shRNA)-mediated HMGB1 downregulation in the post-ischemic brain suppressed infarct size [25]. Reducing HMGB1 expression by shRNA attenuated ischemia-dependent microglia activation and induction of inflammatory cytokines and enzymes (TNF-α, IL-1β and iNOS) in the ischemic brain [25].

More recently, treatment with neutralizing anti-HMGB1 monoclonal antibody (mAb) remarkably ameliorated brain infarction induced by a 2-hour occlusion of the middle cerebral artery in rats, even when the mAb was administered after the start of reperfusion [41]. Furthermore, anti-HMGB1 antibody inhibited the activation of microglia, the expression of TNF-α, and iNOS. In contrast, intracerebroventricular injection of HMGB1 increased the severity of infarction and neuroinflammation [41].

Additional evidence indicating that HMGB1 is associated with ischemic brain injury comes from experiments showing that downregulation of HMGB1 brain levels with rabbit polyclonal anti-HMGB1 antibody correlates with diminished infarct volumes [27].

In patients with ischemic stroke, the serum or plasma levels of HMGB1 are dramatically higher than those in age- and gender-matched controls [27,40]. In an ischemic stroke animal model, the serum level of HMGB1 increased 4 hours after ischemia [21,26], and HMGB1 was massively released into the extracellular space immediately after ischemic insult. HMGB1 subsequently induced the release of inflammatory mediators in the post-ischemic brain [21]. Intriguingly, regarding the relocation dynamics of HMGB1 in the neuronal cells, HMGB1 translocated from the neuron nuclei to the cytoplasm and subsequently was depleted from neurons after one hour of MCAO [26,28], indicating that HMGB1 is released early after ischemic injury from neurons.

Interestingly, one most recent study found that intracerebroventricular injection of recombinant human HMGB1 (rhHMGB1) in TLR4^+/+ ^mice but not in TLR4^-/- ^caused significantly more injury after cerebral ischemia-reperfusion than in the control group, suggesting that TLR4 contributes to HMGB1-mediated ischemic brain injury [20]. Moreover, to determine the potential downstream signaling of HMGB1/TLR4 in cerebral ischemic injury, the ischemic-reperfusion model in TRIF^-/- ^and ^+/+ ^mice were used to evaluate the activity and expression of TRIF pathway-related kinases [20]. There were no obvious differences in ischemic injury between the TRIF^-/- ^and TRIF^+/+ ^mice.

In addition, the protein levels of TANK binding kinase 1 (TBK1), total IKKε, and phosphorylated-IKKε, were determined in TRIF^-/- ^and TRIF^+/+ ^mice. TRIF^-/- ^mice showed no changes in TBK1, total IKKε, and phosphorylated-IKKε in response to ischemia-reperfusion [20]. The results suggest that HMGB1 mediates ischemia-reperfusion injury by TRIF-adaptor independent TLR4 signaling.

However, several basic questions still need to be answered before the broad picture of TLR involvement in cerebral ischemic injury can emerge. So far, studies on TLRs in ischemic brain stroke have mainly focused on ischemic damage in TLR4- and, to a lesser extent, TLR2-mutant mice. Although this approach has provided a first glimpse into the relevance of TLR signaling in ischemic stroke, it has not enabled an understanding of the role of TLR signaling in specific cell types. This issue is of great importance because the pathology of ischemic stroke involves many different cells, e. g., neurons, astrocytes, microglial, endothelial cells, and invading immune cells. More recently, Weinstein et al [42] present new experimental data about genomic microarray analyses on primary mouse microglia derived from either wild-type (WT) or TLR4^-/- ^mice following exposure to either ischemia-reperfusion or control conditions. They found that the markedly disparate genomic responses that occur in wild-type vs. TLR4-/- microglia following exposure to hypoxic/hypoglycemic conditions. These data have provided further molecular insights into both the effect of ischemia on the microglial phenotype and the role of microglial TLR4 in ischemia-induced neuroinflammation and suggested that TLR4 signaling in microglia during ischemic injury play an important role in ischemia-induced inflammatory injury.

### TLRs and cerebral ischemic tolerance

A great amount of evidence from experimental studies supports the detrimental role of innate immunity in cerebral ischemic injury. As we discussed above, ablation of TLR2, 4 and other components of TLR signaling (HMBG1) *in vivo *seems to decrease infarct size, attenuate inflammatory responses, and improve neurological behavior in animal models of cerebral ischemia. Thus, targeting TLR signaling may be a novel therapeutic strategy for cerebral ischemic injury and other inflammatory diseases. For example, stimulation of some TLRs prior to ischemia provides robust neuroprotection. TLR ligands administered systemically induce a state of tolerance to subsequent ischemic injury. The stimulation of TLRs prior to ischemia reprograms TLR signaling that occurs following ischemic injury. Such reprogramming leads to suppression of pro-inflammatory molecules, while numerous anti-inflammatory mediators are enhanced [13].

### The role of TLR4 in ischemic brain tolerance

Pre-exposure of the brain to a short ischemic event can result in subsequent resistance to severe ischemic injury [13], a phenomenon known as preconditioning. Preconditioning ischemic tolerance has been observed in humans in clinical practice. Indeed, less severe strokes have been described in patients with prior ipsilateral transient ischemic attacks within a short period of time [43].

TLR4-induced tolerance to cerebral ischemia was first demonstrated with low-dose systemic administration of LPS, which rendered spontaneously hypertensive rats tolerant to ischemic brain damage induced by MCAO [44]. Since then, LPS-induced tolerance to brain ischemia has been demonstrated in a mouse model of stroke and in a porcine model of deep hypothermic circulatory arrest [44,45].

The exact molecular mechanisms underlying ischemic tolerance are not well understood, but requirements for *de novo *protein synthesis, activation of the proinflammatory transcription factor NF-κB, and induction of inflammatory cytokines such as TNF-α, IL-1β, and IL-6 have been demonstrated [46]. Suppression of the normal inflammatory responses to ischemia is a hallmark of the LPS-preconditioned brain. Administration of low-dose LPS before MCAO prevented the cellular inflammatory response in the brain and blood. Specifically, LPS preconditioning suppressed neutrophil infiltration into the brain and microglia/macrophage activation in the ischemic brain, which was paralleled by suppressed monocyte activation in the peripheral blood [44].

Moreover, preconditioning with LPS protects the brain against the neurotoxic effects of TNF-α after cerebral ischemia [47]. Mice that had been preconditioned with LPS prior to ischemia showed a pronounced suppression of the TNF-α pathway following stroke, with reduced TNF-α in the serum [47]. LPS-preconditioned mice also showed marked resistance to brain injury caused by intracerebral administration of exogenous TNF-α after stroke [47]. Therefore, suppression of TNF-α signaling during ischemia confers neuroprotection after LPS preconditioning

Interestingly, one recent study investigated whether cerebral ischemia induced by MCAO for 2 hours differed in mice that lack functional TLR3 or TLR4 signaling pathways [48]. As a result, TLR4-, but not TLR3-knockout mice had significantly smaller infarct area and volume 24 hours after ischemia-reperfusion compared with wild-type mice [48]. Moreover, ischemic preconditioning induced by a 6-min temporary bilateral common carotid artery occlusion provided neuroprotection, as shown by a reduction in infarct volume and better outcome in mice expressing TLR4 normally but not in TLR4-deficient mice [49]. Mice that have been preconditioned displayed a pronounced reduction of TNF-α, iNOS, and COX-2 in the brains of wild-type TLR4 mice relative to TLR4-deficient mice [49]. Taken together, TLR4 is involved in neuroprotection afforded by ischemic preconditioning.

### The role of TLR9 in ischemic brain tolerance

Recently TLR9 was shown to induce tolerance to brain ischemia [50]. Systemic administration of the immunostimulus CpG-ODN1826 in advance of MCAO reduced ischemic damage up to 60% in a dose- and time-dependent manner [50]. Moreover, pretreatment with CPG protected neurons in both *in vivo *and *in vitro *models of stroke [50]. Notably, the protection afforded by CpG depends on TNF-α, as systemic CpG administration acutely and significantly increases serum TNF-α, and TNF-α knockout mice fail to be protected by CpG preconditioning [50]. Therefore, preconditioning with a TLR9 ligand induces neuroprotection against ischemic injury through a mechanism that shares common elements with LPS preconditioning via TLR4. Additionally, similarities among the known TLR signaling pathways and their shared ability to induce TNF-α suggest that stimulation of TLR4 and TLR9 may induce ischemic tolerance by similar means.

The demonstration that ischemic tolerance in the brain occurs through TLR9, in addition to TLR4, raises the possibility that this is a conserved feature of all TLRs. Recognition that TLR9 is a new target for preconditioning broadens the range of potential antecedent therapies for brain ischemia. Phase II clinical trials are already in progress with CpG-ODNs for use in adjuvant and anticancer therapies [51]. Thus, CpG-ODNs may offer great translational promise as a prophylactic treatment against cerebral morbidity.

### Mechanisms of TLR-induced neuroprotection in cerebral ischemia

Since administration of LPS can induce ischemic tolerance [52], Karikó et al. developed a hypothetic model to explain this phenomenon [52]. They hypothesized that tolerance is dependent on the inhibition of the TLR and cytokine signaling pathways, suppressing in this way the inflammatory response to ischemia [53]. When an ischemic infarction takes place, the resultant cascade of molecular events normally involves TLR activation and cytokine expression, which activates inflammation, among other mechanisms. TLR and cytokine signaling subsequently trigger other pathways that induce immune suppression by increasing signaling inhibitors, decoy receptors, and anti-inflammatory cytokines. Thus, when another ischemic event occurs the presence of inflammatory inhibitors reduces the inflammatory response and subsequent secondary cell death [13,53].

In fact, the finding that TLRs are mediators of ischemic injury provides insight into the potential mechanisms of LPS- and CpG-induced neuroprotection [12,13,47,54]. Cells that are tolerant of LPS are characterized by their inability to generate TNF-α in response to TLR4 activation. Upon TLR4 ligation, LPS-tolerant cells, unlike naïve cells, do not recruit MyD88 to TLR4, and fail to activate IRAK-1 and NF-κB [55]. The TLR4-NF-κB signaling axis becomes decommissioned following a primary exposure to LPS via an elaborate negative feedback loop. This loop involves known inhibitors of TLR signaling, including Ship-1, which prevents TLR4-MyD88 interaction, as well as IRAK-M, a non-functional IRAK decoy, and TRIM30α, which destabilizes the TAK1 complex [56,57]. Thus, subsequent signaling of TLR4 to NF-κB is blocked and inflammatory cytokine production is suppressed. Conversely, it was also found that secondary exposure increased signaling via the TLR4-IRF3 axis and caused enhanced IFN-β release [54]. Thus, pretreatment with LPS causes cells to switch their transcriptional response to TLR4 stimulation, by enhancing the IRF3- induced cytokine IFN-β, and suppressing the NF-κB-induced cytokine TNF-α.

Similar to LPS tolerance, priming TLR9 with CpG induces a state of hyporesponsiveness to subsequent challenge with CpGs [58]. Interestingly, cross tolerance between the two receptors has also been reported, as ligands for TLR9 induce tolerance against a subsequent challenge with a TLR4 ligand [54,59]. CpG-pretreated cells not only produce less TNF-α when secondarily challenged with LPS, they also produce significantly greater levels of IFN-β [54]. This observation suggests that the mechanism of neuroprotection between LPS and CpG preconditioning share common elements.

Therefore, TLR stimulation prior to stroke may reprogram ischemia-induced TLR activation. Specifically, administration of LPS or CpG may activate TLR4 and TLR9, respectively, causing a small inflammatory response, with an initial rise in TNF-α. Cells would then regulate their inflammatory response through expression of negative feedback inhibitors of the TLR4-NF-κB signaling axis, when cells are subsequently exposed to endogenous TLR ligands generated from ischemia-injured tissue. Within this new cellular environment, stimulated TLRs such as TLR4 would be unable to activate NF-κB-inducing pathways. Therefore, stroke-induced TLR4 signaling may be blocked completely, leading to reduced injury, and stroke-induced TLR4 signaling would shift from NF-κB induction to IRF3 induction. Suppression of NF-κB induction would be expected to protect the brain, as mice lacking the p50 subunit of NF-κB suffer less cerebral ischemic damage than wild-type mice [60]. Enhancement of IRF signaling would also be expected to protect the brain, as IFN-β, a downstream product of IRF3 induction, has been shown to act as an acute neuroprotectant [61,62].

### Therapeutic interest in TLRs in cerebral ischemia

Since it has been established that TLR activation after ischemia by endogenous ligands contributes to tissue damage in stroke, the development of therapies that target TLRs and their associated signaling pathways may be useful in the treatment of cerebral ischemia. TLR activation before ischemia has been shown to be protective [13,47,49,50,63].

Indeed, as mentioned above, several lines of evidence suggest that TLR4 is involved in a protective effect induced by preconditioning against ischemic brain injury [13,49,54,63]. TLR4 is involved in ischemic preconditioning where ischemia of short duration provides resistance to subsequent challenge, thus conferring ischemic tolerance [49]. Moreover, pretreatment with the TLR9 agonist CpG before MCAO also conferred neuroprotection [50].

Importantly, one most recent study demonstrated for the first time that pharmacological preconditioning against cerebrovascular ischemic injury is also possible in a nonhuman primate (rhesus macaque) model of stroke[64]. The model of stroke used was a minimally invasive transient vascular occlusion, resulting in brain damage that was primarily localized to the cortex, and as such, represents a model with substantial clinical relevance.

K-type cytosine-guanine-rich DNA oligonucleotides are currently in use in human clinical trials, underscoring the feasibility of this treatment in patients at risk of cerebral ischemia [64]. Finally, another clinical study indicates that preconditioning may occur naturally in humans after transient ischemic attacks and mild strokes [65]. Therefore, as ischemic preconditioning activates endogenous signaling pathways that culminate in protection against ischemic brain damage, drugs that stimulate TLRs might protect against cerebral ischemic injury.

On the other hand, it has also been proposed that HMGB1, an exogenous ligand of TLRs, protects against cerebral ischemic injury [30]. For example, there is evidence that HMGB1 antibodies improved the outcome in an animal model of stroke [27,41,66]. Moreover, in a mouse model of cerebral ischemic stroke, systemic administration of HMGB1 box A protein significantly ameliorated ischemic brain injury [27], suggesting that HMGB1 box A may provide a tool for therapy. However, to date, the use of HMGB1 as a pharmacologic treatment in clinical cerebral ischemic injury has not been explored.

### Conclusions and prospective

Ischemic brain injury after cerebral ischemia results from a complex pattern of pathophysiological events. The contribution of inflammation to ischemic neuronal damage is well known. TLRs are critical components of the innate immune system that have been shown to mediate ischemic injury. So far, there have only been a few studies that examine the role of TLRs in cerebral ischemia, and some of them suggest that TLRs are involved in the enhancement of cell damage following ischemia [23,24,36]. TLR2 and TLR4 and their ligand HMGB1 have been well documented to contribute to ischemic brain damage [12,23,32-34,36,38].

The activation of TLR signaling leads to ischemic preconditioning [12,13,34,47,50]. Recently, TLR4 and TLR9-induced tolerance to cerebral ischemia has been well studied. The stimulation of TLR4 and TLR9 may induce ischemic tolerance by similar means. LPS preconditioning reprograms the cellular response to stroke, which may represent endogenous processes that protect the brain against additional injury.

By setting the stage for improved ischemic outcome, TLR reprogramming offers a low-risk, high-benefit opportunity to combat neuronal injury in the event of cerebral ischemia [64]. CpG appears to be a unique preconditioning agent, coordinating both systemic and central immune components to actively protect the body from cerebral ischemic injury.

## List of abbreviations used

TLR: toll-like receptor; CNS: central nervous system; BBB: blood-brain barrier; ER: endoplasmic reticulum; PAMP: pathogen-associated molecular patterns; LPS: lipopolysaccharide; HMGB1: high mobility group box 1 protein; MCAO: middle cerebral artery occlusion; iNOS: inducible nitric oxide synthase; COX2: cyclooxygenase 2; MyD88: myeloid differentiation primary response gene 88; MAL: MyD88 adaptor-like protein; TRIF: TIR-domain-containing adaptor protein inducing interferon (IFN)-β-mediated transcription factor; TRAM: TRIF-related adaptor molecule; SARM: sterile α- and armadillo motif-containing protein; IRAK: IL-1 receptor associated kinase; IRF: interferon-β promoter-binding protein; TBK1: TANK binding kinase 1

## Competing interests

The authors declare that they have no competing interests.

## Authors' contributions

WYC collected literatures and reviewed the literatures. LS reviewed the literatures and proofread and corrected the manuscript. YQW wrote the manuscript and has approved the final version of the manuscript. All authors read and approved the final manuscript.
